# Understanding mealtime behavioral problems in nursing home residents living with dementia: a group concept mapping approach

**DOI:** 10.1186/s12877-024-05420-4

**Published:** 2024-10-16

**Authors:** Eline C.P. van Buuren, Marieke Perry, Christian Bakker, Raymond T.C.M. Koopmans, Jenny T. van der Steen

**Affiliations:** 1 Department of Primary and Community Care, Radboud university medical center, Geert Grooteplein Zuid 21, 6525EZ, Nijmegen, The Netherlands; 2 ‘Joachim en Anna’, center for Specialized Geriatric Care, Stichting De Waalboog, Groesbeekseweg 327, 6523PA, Nijmegen, The Netherlands; 3 Radboudumc Alzheimer Center, Geert Grooteplein Noord 15, 6525EZ, Nijmegen, the Netherlands; 4 Department of Public Health and Primary Care, Leiden University Medical Center, Hippocratespad 21, 2333ZD, Leiden, The Netherlands; 5 General Medical Practice, Rozendaalselaan 34, 6881LD, Velp, The Netherlands; 6 Groenhuysen, Center for Geriatric Care, Bovendonk 29, 4707ZH, Roosendaal, The Netherlands

**Keywords:** Concept mapping, Dementia, Behavioral problems, Nutrition, Hydration

## Abstract

**Background:**

Persons with dementia frequently experience mealtime behavioral problems that can result in reduced or lack of intake of food or fluids. Multiple underlying causes and expressions of mealtime behavioral problems complicate its interpretation and intervention, because problems originating from cognitive and functional decline and behavioral changes may interact. Healthcare professionals and family caregivers may encounter a variety of practical and moral dilemmas in dealing with these problems. We aimed at a better understanding of mealtime behavioral problems and related complex issues in nursing home residents with dementia from a daily practice perspective.

**Methods:**

We used a mixed-method Group Concept Mapping approach in this study, and collected data online with a panel of 67 healthcare professionals, researchers and relatives from across The Netherlands. The participants contributed to either or all of the following phases: (1) the generation of ideas (brainstorm), (2) sorting, and (3) rating of the ideas. Subsequent phases included data analysis with Groupwisdom^®^ software and interpretation of the results. Multidimensional scaling and hierarchical cluster analysis resulted in a concept map visualizing the coherence and importance of ideas. Bridging values were calculated, with low values indicating a distinct, clear concept.

**Results:**

Brainstorming resulted in 285 statements representing 85 ideas. The concept map visualized three categories capturing ten clusters which describe the management of mealtime behavioral problems, causes of mealtime behavioral problems, and expressions and interpretations of mealtime behavioral problems. Concepts reflecting direct consequences, ethical components, and considerations to handle challenging situations overlapped on the concept map with the highest bridging values (range 0.58–0.87).

**Conclusion:**

This study added to unraveling the complex nature of mealtime behavioral problems, as perceived in practice. It is recommended to comprehensively analyze all components in the management of these problems, in particular being aware of ethical factors and align care for residents with dementia accordingly.

## Introduction

Mealtime behavioral problems often occur in persons with dementia, and can cause a critical status of nutrition and hydration when these problems impede the intake of food and fluids [1–5]. In this study, we refer to mealtime behavioral problems as any behavior that may indicate resistance of residents with dementia to eat or drink, not exclusively observed during mealtime. Mealtime behavioral problems occur in any type of dementia but express differently across the phases of dementia [6, 7]. Previous studies have mainly focused on older persons with dementia, but appetite and eating changes are reported among the most common behavioral changes in persons with young-onset dementia as well [8]. Therefore, in this study we consider a broad spectrum of dementia, regardless of age of onset and dementia type.

Malnutrition is a common consequence of mealtime nutritional problems, and the prevalence rates of malnutrition resulting from insufficient nutritional intake vary [5, 9]. A recent meta-analysis reports 32.5% of older adults with dementia being malnourished, and 54.7% being at risk of malnutrition. In general, prevalence rates increase in persons who are diagnosed with dementia and reside in a long-term care setting, and rise even further with increasing care needs [9]. Poor nutritional intake may lead to dehydration as well, and is reported in 0.8-38.5% of nursing home residents [10]. Adverse outcomes of mealtime behavioral problems further include reduced quality of life, aspiration pneumonia, and an increased mortality risk [5, 11–14].

Mealtime behavioral problems are caused by a variety of factors [15, 16]. Physical and cognitive changes associated with dementia result in functional decline and may lead to increased difficulties with expressing needs, greater dependency upon others during meals, and problems such as apraxia [1, 5]. Deterioration of sensory capabilities may lead to an altered sense of taste and smell, influencing the experience of eating and drinking [1]. In addition to functional problems originating from physical and cognitive decline, behavioral symptoms may relate to, display or exacerbate at mealtimes [16, 17].

Behavioral changes that result from the dementia and complicate the intake may be expressed during the mealtime by throwing food or cutlery, refusing to eat or leaving the table [1, 11, 18]. Such behavioral changes can be perceived as challenging by professionals and relatives who assist persons with dementia during mealtimes. They may also encounter moral dilemmas when situations around intake of food and fluids lead to severe malnutrition and decisions on dietary interventions need to be made [2, 14, 19].

The variety of underlying causes and expressions of mealtime behavioral problems complicate their interpretation and related interventions, also because problems originating from physical and cognitive decline, and behavioral changes often interact [3, 16, 18].

Our study aimed to improve the understanding of complexity of mealtime behavioral problems in persons with dementia. We describe and define these issues to better unravel and collaboratively manage these problems in daily practice.

## Materials and methods

### Study design

In this study, a group concept mapping approach was used to map mealtime behavioral problems that impede the intake of nutrition and hydration in nursing home residents with dementia.

Group concept mapping is a mixed-method approach which combines quantitative and qualitative research methods to map ideas of a group of participants [20]. The input of participants enables researchers to clarify a complex topic [21]. The approach has been used in various contexts, including health care. Group concept mapping consists of five phases and Table 1 provides an overview of the actions, actors and results of Group Concept Mapping per phase. Data was collected online using Groupwisdom^®^ [22]. The participants contributed to the data collection between November 2021 and February 2022 (phases 2 and 3). Data analysis and interpretation (phases 4 and 5) took place from March 2022 until November 2022. The researchers analyzed and prepared each step of data collection.

**Table 1:** Description of the phases of the group concept mapping

Study phase	Actions	Actors	Results
Phase 1: preparation September - October 2021	- Writing a concept mapping plan - Developing a focus prompt - Pilot test of the focus prompt with five healthcare professionals - Adjustment of the focus prompt	EvB EvB, MP, JvdS, CB, RK EvB EvB, MP, JvdS, CB, RK	- Concept mapping plan - Focus prompt (to guide data collection) - Final focus prompt
Phase 2: generating the ideas (brainstorming activity) November – December 2021	- Sharing individual ideas by completing the focus prompt in online individual brainstorm session - Idea analysis after brainstorm session: reducing the statement list by removing identical statements, splitting in case of > 1 idea, combining overlapping statements	69 participants EvB, MP, JvdS	- 285 statements - Final statement list of 85 ideas resulting from idea analysis
Phase 3: structuring of statements (sorting and rating activity) January – February 2022	- Structuring 85 statements in piles and providing labels - Rating 85 statements on importance - In- and exclusion of datasets for data analysis	41 participants 40 participants EvB, MP, JvdS	- 33 sorting and rating data sets included in data analysis
Phase 4: analysis (representation of ideas in concept map) March-June 2022	- Choosing a scenario for data analysis - Multidimensional scaling - Hierarchical cluster analysis	EvB, MP, JvdS	- Final scenario based on data sets of 33 participants who completed both the sorting and rating activity - Point map visualizing 85 ideas - Concept map describing 10 clusters based on the 85 ideas - Cluster rating map describing the lowest and highest rates (both on statement level and cluster level)
Phase 5: interpretation June-November 2022	Discussing the results	EvB, MP, JvdS, CB, RK, expert group	Final labels and descriptions of the concepts on the cluster map

### Sampling and participants

In group concept mapping, the aim is to capture a broad perspective of the subject, and therefore the participants need to be selected carefully [23]. We purposefully sampled persons we considered experts on the subject of this study, and approached healthcare professionals, researchers, and relatives of persons with dementia. Participants were eligible when meeting the criteria of (1) having experiential or theoretical knowledge of mealtime behavioral problems impeding intake of nutrition and hydration in persons with late onset or young onset dementia, and (2) having some digital skills, because of online data collection. We aimed to include 100 participants to ensure a variety of input from participants with different backgrounds. In this study, we refer to young-onset dementia when the onset of the dementia was below the age of 65 years and to late-onset dementia when diagnosed at the age of 65 or over [24]. Healthcare professionals and relatives were recruited from nursing homes affiliated with the University Knowledge Network of Older Adult Care Nijmegen (UKON) and the Young-onset Dementia Knowledge Center, the Netherlands. Researchers were approached via the network of the research team, and developers of the current Dutch guideline on this topic were recruited as well [25]. No incentives were used for recruitment and participants did not receive financial compensation.

### Data collection and analyses

#### Phase 1: preparation

The process of group concept mapping starts with a central problem or question, that is translated into a focus prompt to guide the content of the data collected. The focus prompt we developed was: *‘When I think of mealtime behavioral problems in people with dementia*,* I think of…’.* It was pilot tested by five healthcare professionals, which resulted in a quite broad but relevant range of statements. We maintained the formulation in order to generate a comprehensive range of ideas. The participants received instruction per email to create an online Groupwisdom account and gave their consent online. Subsequently, they were asked to provide the following demographic characteristics: gender, profession, and years of experience with mealtime behavioral problems, target group of experience (young-onset dementia, late-onset dementia, or both) and type of experience (role as relative, clinical, theoretical). All participants who registered and gave their consent were invited to at least the generation of ideas (brainstorming activity), but also to contribute to the structuring of statements (sorting and rating activity).

#### Phase 2: generating ideas (brainstorm)

In a brainstorming activity, participants were asked to complete the focus prompt. The participants could share as many ideas as they wished. After closing the brainstorm activity, researchers EvB, JvdS, and MP reduced the statement list by removing duplicates and merging similar content. For example, statement 41 was merged with 10 other statements that contained the same content, varying from pushing away a plate, shoving a plate away, pushing a hand away, making a gesture of rejection etc. The duplicate statements were removed and reformulated into one statement that captured all examples with a similar meaning. Further, statements that contained multiple ideas were split; statement 48 was split into not wanting to eat, and not being hungry. Lastly, statements were reformulated to improve clarity. For example, statement 249 ‘A way for the person with dementia to maintain some control’ was reformulated to ‘That defensive behavior is a way for the resident to retain some sense of control’. We aimed at a maximum of 100 statements as recommended in literature [23, 26].

#### Phase 3: structuring of statements

The structuring of statements consisted of a sorting and importance rating activity. For the sorting activity, participants reviewed the final statement list, created piles with statements they considered to represent similar concepts, and formulated a label for each pile. Subsequently, participants were asked to rate the statements on a 5-point Likert-scale, ranging from not important at all to very important [1–5]. The results were reviewed by EvB, JvdS, and MP in order to determine what entries of individual participants could be included for data analysis. A minimum of 75% completion of both the sorting and rating task was maintained for inclusion in data analysis. Additional criteria that we used for reviewing the sorting data sets were minimal coherence of the statements in the piles, and an average of 10–15 piles per participant [23]. Raw sorting and rating results were checked by EvB, and in case of doubt, the data set was discussed with JvdS and MP, before reaching consensus on a final decision. We included 33 data sets for data analysis from participants who both completed the sorting and rating activity.

#### Phase 4: data analysis

In data analysis, a similarity matrix was formed based on the 33 data sets from the sorting task. This matrix reflects the number of participants that sorted each pair of statements together. Next, multi-dimensional scaling was applied which resulted in a point map visualizing how statements were sorted based on the similarity matrix. Statements that were sorted together frequently in a pile are depicted close proximity on the point map. The amount of points represent bridging values (range 0–1); the lower the value, the more defined the concept of a group of statements is. A high bridging value implicates a less defined concept, because in this case the statement was linked to statements that are more distant on the point map. Further, a stress value that represents the goodness of fit was calculated for the point map. A high stress value indicates discrepancy between the similarity matrix and visualization of the data in the point map. The cut-off value in group concept mapping is 0.39 [23, 26, 27]. Subsequently, hierarchical cluster analysis was applied, based on the positions of the statements on the point map along with the bridging values. Hierarchical cluster analysis results in a transformation on another level, zooming out from individual statements to conceptual themes (or clusters). This is visualized via a cluster map capturing the concepts and suggesting labels for the overarching concepts based on the labels provided by the participants during the sorting activity. To decide about a cluster map solution, we assessed a range of 3–20 clusters as recommended [23]. The basic principle in hierarchical cluster analysis is to arrange clusters in a hierarchical tree structure and choose a cluster solution that is the most desirable for interpretation of the concept being studied. Because the group concept mapping approach does not provide specific techniques or actions to carefully handle possible influence of the research team [28], we employed standards for analyses of qualitative data. Researchers EvB, JvdS, and MP individually examined and merged the clusters in a stepwise manner, followed by comparing proposed adaptions and solutions, and discussing them until reaching consensus. This resulted in the selection of a concept map with 10 clusters that described mealtime behavioral problems in the most detail.

#### Phase 5: interpretation of the concepts

The proposed concept map was discussed by the research team and an independent expert group that was involved with the study, resulting in a final concept map. The expert group consisted of an independent panel of various professionals and caregivers that advised the research team. The expert group members did not participate in data collection.

## Results

### Participant and process characteristics

A total of 93 participants out of 14 nursing homes were assigned to Groupwisdom^®^. The response rate for the brainstorming activity was 72%, for the sorting activity 44%, and for the importance rating activity 43%. Most participants (85%) were female and most (90%) were healthcare professionals (Table 2). The participants had a mean of 12.5 (9.4) years of experience in dealing with mealtime behavioral problems in people with dementia, and most participants (58%) had knowledge of problems related specifically to older people with dementia (Table 2).

**Table 2:** Participant characteristics

Participant characteristics *N* = 67 (72%)	
**Sex (%)**	
Male Female Other	9 (13%) 57 (85%) 1 (2%)
Role (%)	
Relative Researcher Healthcare professional	3 (4%) 4 (6%) 60 (90%)
- *Nursing staff* - *Care staff* - *Nurse* - *Psychiatric nurse* - *Kitchen/mealtime support staff)* - *Physician* - *Psychologist* - *Speech therapist* - *Dietician* - *Occupational therapist* - *Physiotherapist* - *Spiritual counsellor)*	*16 (26%)* *5 (8%)* *7 (12%)* *2 (3%)* *2 (3%)* *12 (20%)* *9 (15%)* *8 (13%)* *5 (8%)* *5 (8%)* *4 (7%)* *1 (2%)*
Years of experience, mean (SD), range	12.5 (9.4), 1–45
Knowledge and experience related to^1^	
People with young-onset dementia People with late-onset dementia Both people with young- and late-onset dementia	6 (9%) 38 (58%) 22 (33%)

1. 1 missing

**Fig. 1:**
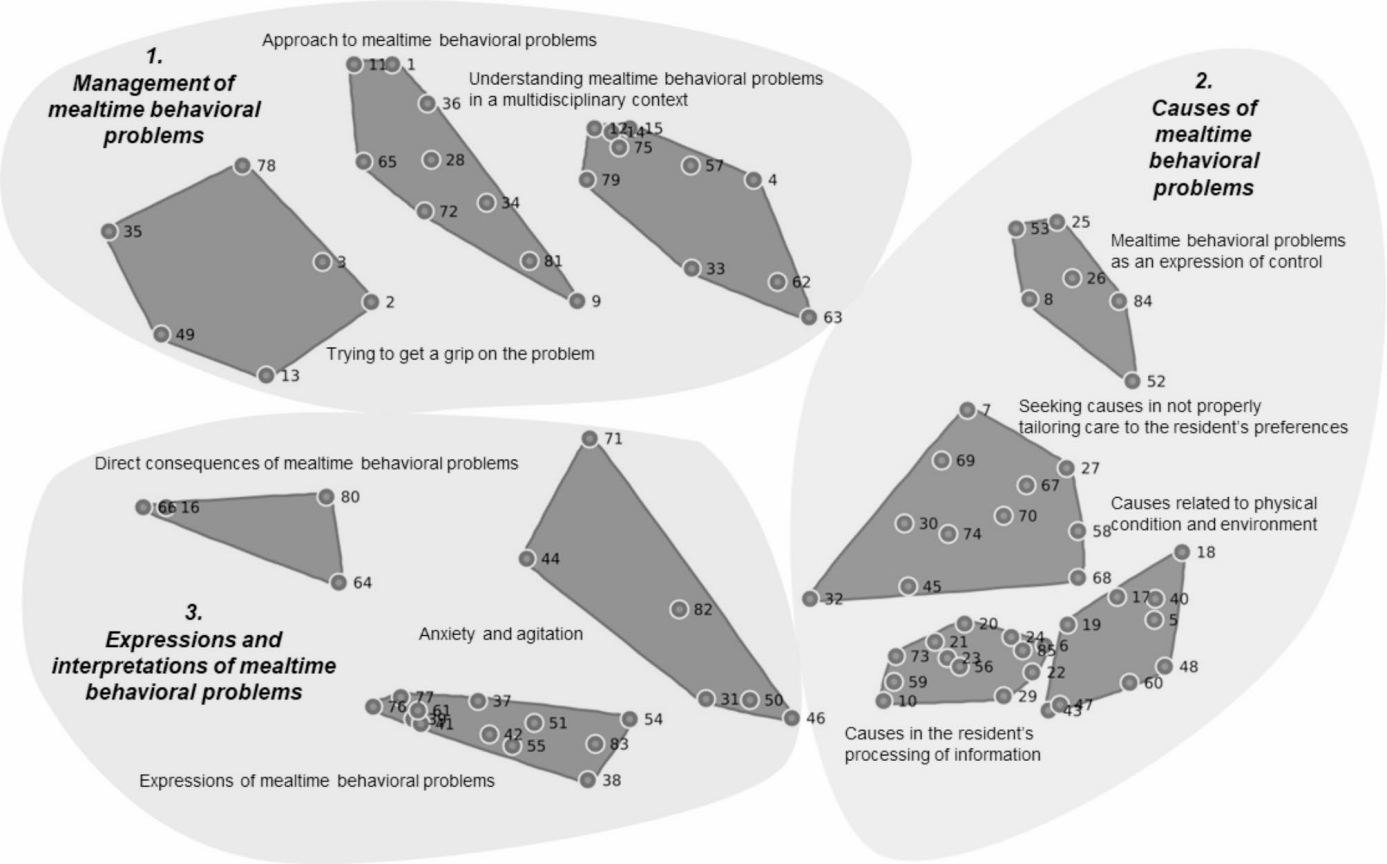
Final concept map representing mealtime behavioral problems. Legend: Visualization of 85 statements in ten clusters which describe (**1**) the management of mealtime behavioral problems, (**2**) causes of mealtime behavioral problems, and (**3**) expressions and interpretations of mealtime behavioral problems

The focus prompt was completed 285 times by 67 participants who formulated one or more statements in the brainstorm activity (phase 2). The sorting activity was completed 41 times and the rating activity 40 times, of which 33 data sets were included for data analysis. Multidimensional scaling using the sorting data resulted in a point map. In hierarchical cluster analysis, we agreed upon a final concept map of 10 clusters comprising statements regarding mealtime behavioral problems (Fig. 1, final concept map). The stress value of the concept map was 0.26 (range 0–1), indicating a high goodness of fit and with it sufficient representational validity. An overview of the clusters with descriptions along with the statements per cluster is provided in Table 3. The clusters and statements are sorted in ascending order of bridging values.

**Table 3:** Overview of clusters and description of the content including mean bridging and rating values

Cluster label, (bridging value), and description of the content^1^	Statements
**Causes in the resident’s processing of information (0.05)** *Causes of mealtime behavioral problems resulting from altered stimulus processing*,* and causes resulting from altered functioning due to the dementia.*	23 Apraxia (difficulty performing particular movements, such as using cutlery). 0.00 56 Not being aware of feeling hungry, not being hungry (anymore). 0.00 73 That the resident does not recognise the food or cutlery. 0.01 20 Reduced speed of information processing of the resident. 0.02 21 That a resident is occupied with something other than eating at mealtime. 0.02 24 Resident’s decreased appetite. 0.05 59 That the resident with dementia is unable to clearly articulate his/her opinions or thoughts. 0.05 85 That the resident is too tired to eat. 0.05 29 Altered sensation in the mouth. Examples: the fork/spoon does not feel comfortable, the resident does not like the consistency of the food. 0.06 10 Decreased comprehension of the resident with dementia. 0.07 22 That a resident does not like the smell or taste of the food. 0.10 6 Depression. 0.13
**Causes related to physical condition and environment (0.21)** *Causes of mealtime behavioral problems that are medical in nature or stem from an unsuitable environment. These are external to the resident*,* who has no control over them*,* but is affected by them.*	19 Physical complaints, such as abdominal pain or nausea 0.08 43 Oral pain. 0.11 47 Dental problems. 0.11 5 Whether the resident is experiencing pain. 0.21 60 Under-stimulation, for example due to lack of atmosphere and engagement during the meal. 0.24 17 Swallowing problems. 0.25 18 The final stage of life, which results in a slow loss of appetite. 0.36 40 Side effects of medication. Examples: dry mouth, altered taste, fungal infection, or drowsiness. 0.25 48 Too many stimuli in the environment during mealtimes, causing the resident to, for example, become distracted. 0.28
**Seeking causes in not properly tailoring care to the resident’s preferences (0.24)** *Looking for causes of mealtime behavioral problems in how the resident deals with eating and drinking and with the situation at mealtimes*,* and whether care is properly attuned to the resident’s preferences.*	45 Offering food in an unsuitable way. Examples: too fast, at the wrong time, pace too high, the person assisting is not calm enough, style of communication is not in line with resident needs. 0.09 32 Refusing food because the resident does not want to, is unable to or no longer understands how to eat and drink. 0.16 74 Whether the conditions for eating are present, such as whether the resident is awake/rested. 0.16 68 The sitting position. 0.21 70 Whether there is a negative experience with food and drink in general or with a specific dish. 0.23 58 Resident inadequately prepared for the meal, for example, [therefore] incomprehension, no smell of food, [no] quiet environment. 0.24 27 Whether there is unwillingness or ignorance on the part of the resident. 0.36 30 Not wanting to eat anymore. 0.26 69 The entire situation at the mealtime, such as: where does the resident sit in the living room/bedroom, table companions, commotion in the room due to, e.g., radio/TV or people talking. 0.26 67 Whether the resident likes the food. Examples: is this what the resident is used to eating, did he/she like this before, the food is too bland/too heavily seasoned, the resident does not like the consistency. 0.28 7 Whether there is a desire not to live anymore. 0.39
**Expressions of mealtime behavioral problems (0.30)** *Examples of how mealtime behavioral problems can be expressed by a person with dementia*	54 Anger. 0.22 42 Throwing cutlery or tableware. 0.23 55 Playing with the food. 0.26 83 Agitated behaviour. 0.27 51 Frustration. 0.30 37 Biting off, chewing the food well and then spitting it out. 0.31 39 Turning away or lowering the head when food or drink is offered. 0.32 41 Making a gesture of rejection with the hands, such as pushing food/drink/cutlery away, shoving the plate away or pushing the caregiver’s hand away. 0.32 38 Letting food or drink drip from mouth. 0.33 61 Using words/phrases indicating rejection when offered food or drink, such as ‘leave me alone’. 0.33 77 Hitting. 0.36 76 Keeping mouth closed when offered food. 0.42
**Anxiety and agitation (0.33)** *Anxiety and agitation related to mealtime behavioral problems resulting in uncomfortable situations*	46 Not calm enough to eat or remain seated at the table, getting up and walking away from the Table 0.19 50 Anxiety. 0.19 31 Discomfort. 0.28 82 That the resident will not accept help. 0.28 71 That it is defensiveness, not aggression. 0.48 44 Risk of choking, with fear of aspiration. 0.58
**Approach to mealtime behavioral problems (0.36)** *Approach to mealtime behavioral problems from the healthcare professional’s perspective*,* including considerations and policy regarding eating and drinking and involving loved ones / family members*	11 Supporting and providing information to loved ones / family members. 0.23 1 Discussing eating and drinking policies with loved ones / family members. 0.24 36. Discussion about initiating tube feeding. 0.26 28 When you stop offering food and drink. 0.35 34 The need for suggestions to deal with mealtime behavioral problems. 0.35 72 That there is no point in pushing; accept that this is (sometimes) part of it. 0.44 65 Accepting defensive behaviours as the dementia progresses. 0.47 81 That eating and drinking is important for residents with dementia, from every perspective. 0.47 9 Whether there is something to be gained from attention to the way in which food and drink are offered. Examples: presenting multiple choices, patience and time of the person offering, going along with the experience of the resident with dementia. 0.48
**Understanding mealtime behavioral problems in a multidisciplinary context (0.37)** *A multidisciplinary approach to jointly identify the cause of mealtime behavioral problems and use an appropriate intervention within the legal framework.*	15 Multidisciplinary collaboration. 0.26 75 That it is problem behaviour requiring a multidisciplinary approach/analysis based on a biopsychosocial explanatory model. 0.26 14 Consultation of specific discipline(s). 0.27 79 How I can better understand or reduce the defensive behaviour 0.34 57 Identifying the cause, as a multidisciplinary team in collaboration with family. 0.36 33 The importance of finding the cause of defensive behaviours. 0.40 4 A comprehensive analysis of the cause of the resident not eating. Examples: a physical or psychological cause. 0.43 62 What it means when the resident says ‘no’ when food or drink is offered. 0.44 12 The Compulsory Care Act (Wzd). 0.45 63 That defensive behaviour does not automatically mean someone does not want to eat or drink something. 0.45
**Mealtime behavioral problems as an expression of control (0.58)** *The resident’s degree of control/being in charge and autonomy* .	8 Whether refusing to eat is a conscious choice. 0.54 52 Loss of control and of sense of being in charge. 0.54 26 Resident expresses autonomy. 0.57 53 That a sense of being in charge is important. 0.59 84 That defensive behaviour is a way for the resident to retain some sense of control. 0.60 25 To what extent the resident is in control. 0.62
**Trying to get a grip on the problem (0.76)** *Challenges regarding the problem of reduced intake and feelings of not being understood and being powerless on the part of family members/ loved ones and care professionals (do we do something about the behavior itself or about nutrition)*	2 Preventing deficiencies by enriching food. 0.59 3 Thinking about what products a resident does take in. 0.61 78 Family willing to do whatever it takes to stimulate intake 0.66 49 Feeling of helplessness of the person who assists in feeding 0.84 35 Lack of understanding on part of family 0.89 13 Compulsion. 0.97
**Direct consequences of mealtime behavioral problems (0.87)** *Possible direct consequences of defensive behaviours in the form of reduced intake and resulting weight loss and dehydration*	64 An umbrella term for behaviour resulting in impaired fluid and nutritional intake. 0.68 80 That many residents with dementia eventually show Alzheimer cachexia (extreme emaciation). 0.83 16 Reduced intake that may cause weight loss, inadequate fluid and/or nutritional intake or malnutrition. 0.95 66 Risk of dehydration. 1.00

^1 The clusters and according statements per cluster are arranged in ascending order of bridging values^

### Interpretation of the concept map

The final concept map represents 85 statements divided into 10 different clusters. The average bridging value on cluster level was 0.41 (range 0.05–0.87), and the average importance rating value was 3.51 (range 3.06–3.78). The three clusters regarding causes of mealtime behavioral problems had the lowest bridging values, which indicates that these are the most defined concepts. *Direct consequences of mealtime behavioral problems* had the highest bridging value, implying that this cluster is not well defined. Overall, three overarching categories can be considered, capturing the management, causes, and expressions and interpretations of mealtime behavioral problems. We initially did not consider solutions with just a few clusters because we anticipated loss of nuance in capturing a complex phenomenon such as mealtime behavior. The 10-cluster solution provided sufficient detail to represent the findings of this study in a final concept map which we regard the main result. Next, we experienced that in grouping clusters to facilitate an integrated discussion, three groups of clusters allowed for a coherent interpretation and discussion. We then decided to empirically test how closely our grouping would resemble the software’s 3-cluster solution. It matched fully without any of the statements moving between clusters and this legitimizes visualization of the concept map with 10 clusters within the three larger concepts. We describe these three larger concepts (categories) and their meaning below.

#### Management of mealtime behavioral problems

The clusters ‘*Approach to mealtime behavioral problems’*,* ‘Trying to get a grip on the problem’*, and *‘Understanding mealtime behavioral problems in a multidisciplinary context’* describe the search for underlying factors and challenges in interpretation of the problems. Several statements illustrate the complexity and considerations in this search, reflected by ‘When you stop offering food and drinks’ (Statement 28) and ‘How I can better understand or reduce the mealtime behavioral problems’ (Statement 79). Further, these clusters include personal experiences regarding the management of mealtime behavioral problems, as expressed in statement 49: ‘Feeling of helplessness of the person who assists in feeding’. Various statements describe the necessity of multidisciplinary collaboration and involvement of family, such as the consultation of specific discipline(s) (Statement 14), and the importance of identifying the cause of defensive behaviors, as a multidisciplinary team in collaboration with family (Statement 33 and 57). The cluster *understanding mealtime behavioral problems in a multidisciplinary context* was considered of the highest importance by the participants (3.98), as were the individual statements in this cluster (Table 3). On the contrary, the cluster *Trying to get a grip on the problem* was given the lowest importance rating of all clusters (3.07). This cluster also had the second highest bridging value on cluster level (0.76), which means that the statements in this cluster were often linked to other clusters by participants. For example, the statement with the highest individual bridging value (0.97) was Compulsion (Statement 13), which was not only sorted in *Trying to get a grip on the problem*, but also frequently linked to *Mealtime behavioral problems as an expression of control* and *Understanding mealtime behavioral problems in a multidisciplinary context.*

#### Causes of mealtime behavioral problems

*Causes related to physical condition and environment*,* Causes in resident processing of information*, and *Seeking causes in not properly attuning care to the resident’s preferences* comprise concepts regarding the search for causes and the variety of these causative factors. Participants distinguished between factors that are related closely to the resident such as (*Causes in resident’s processing of information)*, causes connected to the person who is involved at mealtimes (*Seeking causes in not properly attuning care to the resident’s preferences)*, and more indirect factors (*Causes related to physical condition and environment). Causes in resident’s processing of information* had the lowest bridging value on cluster level, which indicates a relatively well defined concept and a high level of agreement on the collection of statements in the cluster. These direct causes result from altered stimulus processing and/or functioning due to the dementia, such as inability to clearly articulate opinions or thoughts (Statement 59), not recognizing food or cutlery (Statement 73), or causes such as apraxia (Statement 23). The concept that describes causes in the resident’s processing of information was the most defined, which was reflected in the lowest bridging value on both cluster- (0.05) and statement level (0.00-0.13). This indicates that participants consider mealtime behavioral problems resulting from altered stimulus processing and functioning due to dementia a clear concept. Moreover, the other clusters that describe possible causes (*Causes related to physical condition* and *Seeking causes in not properly attuning care to the resident’s preferences)* have low bridging values as well, which illustrates that (the search for) the underlying mechanisms of mealtime behavioral problems also is a well-defined concept.

#### Expressions and interpretations of mealtime behavioral problems

*Expressions and interpretations of mealtime behavioral problems* illustrates various examples of the problems as seen in clinical practice, for example making a gesture of rejection with the hands, such as pushing food, drinks or cutlery away, shoving the plate away or pushing the caregiver’s hand away (Statement 41). The bridging values of the statements are relatively low (range 0.22–0.36), which shows agreement of participant on these examples of the behavior as observed in clinical practice. *Direct consequences of mealtime behavioral problems* had the highest bridging value of all clusters (0.87), indicating that the statements in this cluster were linked the most frequently to the other clusters of the concept map. Risk of choking (Statement 44), and discomfort (Statement 31) are examples of statements that are included in the cluster *Anxiety and agitation*. It was mentioned that mealtime behavioral problems imply defensiveness, and not aggression (Statement 71). *Mealtime behavioral problems as an expression of control* includes statements of the resident’s autonomy and the question of being in charge. This is visualized in Statement 84, for example, which describes mealtime behavioral problems as a way to retain some sense of control for the resident. Examples of expression of control by the resident are also mentioned in *Seeking causes in not properly tailoring care to the resident’s preferences*, reflected by whether there is a desire to not live anymore (statement 7), unwillingness or ignorance (statement 27), and refusing food because the resident does not want to eat (statement 30 and 32). The similarity in examples explains why *mealtime behavioral problems as an expression of control* and *Seeking causes in not properly tailoring care to the resident’s preferences* are positioned close together on the concept map. Overall, the bridging values on both cluster and statement level are average (range: 0.54–0.62), implying that expression of control is a somewhat defined concept.

## Discussion

Using the group concept mapping method, this study identified three categories capturing ten concepts that describe mealtime behavioral problems in persons with dementia, (1) management of mealtime behavioral problems; (2) causes of mealtime behavioral problems; and (3) expressions and interpretations of mealtime behavioral problems. The low bridging values of the three clusters about causes of mealtime behavioral problems (category 2) indicate this is a relatively well-defined category, other categories were less well-defined. Statements that refer to ethical factors emerged in all three categories of the concept map; not only in the third category (e.g. Not accepting help), but also in the first (e.g. When you stop offering food and drink), and second category (e.g. Whether there is a desire to not live anymore). This implies that in the reasoning of healthcare professionals these factors tend to be overlooked or not addressed as an important issue of its own. This study provides insight in the way healthcare professionals view the coherence and interplay between factors around mealtime behavioral problems.

Our study emphasizes the complex nature of mealtime behavioral problems, given that the clusters do not all reflect well defined concepts. Mealtime behavioral problems are complex and dynamic, because various factors are interrelated, complicating the analysis of the problems and care demands. Hodiamont et al. confirm this finding, stating that every care situation is unique and requires different knowledge [29]. Corazza et al. suggest that a different attitude towards clinical complexity, such as mealtime behavioral problems, is needed. When only separate components of clinical problems are analyzed, the present interactions between factors are not considered, leading to misunderstanding and not properly attuned care. Corazza et al. recommend that both biological and non-biological (e.g. environmental, socioeconomic, cultural and behavioral) factors should be considered in complex clinical problems, so that the interventions are tailored to the resident’s needs [30].

The search and considerations to analyze and interpret mealtime behavioral problems and to find a way to manage the problems are reflected in the clusters of the concept map. Several clusters had high bridging values, and therefore were frequently linked to other clusters. A possible explanation could be that participants had some overarching considerations and sorted the statements in the context of linking causes and consequences, rather than labeling it as a separate concept. Although ethical dilemmas were mentioned, like autonomy of the resident and issues regarding treatment decisions, these statements were spread across the concept map and therefore the participants may have considered these dilemmas as part of a more functional problem, rather than an entity of its own. However, it is known that mealtime behavioral problems can affect both healthcare professionals and relatives [31, 32]. Prior research mentions distress, anger and depression in relatives of persons with dementia [33, 34]. In our study, discussions between healthcare professionals and family about treatment, such as stopping to offer food and drinks or initiating tube feeding, were mentioned. Barrado-Martin et al. confirmed this finding, mentioning that situations around mealtime behavioral problems sometimes even lead to conflicts or disagreement [2]. In these situations a moral case deliberation can be helpful to come to a shared decision [35, 36]. Moreira et al. [37] found considerable levels of burden, even with a short time of disease and few changes in the resident’s diet. Because persons involved consider these problems as challenging and burdensome, this stresses the need to understand and properly manage mealtime behavioral problems in clinical practice.

The presence of three different clusters describing possible causes of mealtime behavioral problems implies that participants think it is important to unravel different types of causes. These findings are supported by previous research that found correlations between environmental factors and cognitive function on food intake difficulties in residents with dementia, such as distraction by a turned on radio or television [38]. Jung et al. described in a scoping review that cognitive and physical function, close relationships with family and caregivers, and physical environment are the highest contributing factors to mealtime behavioral problems [3]. Similar factors are mentioned by Fostinelli et al., further adding cultural norms and values as an influencing factor [39]. The concept *Seeking causes in not properly tailoring care to the resident’s preferences* describes how the resident deals with eating, drinking, and the situation at mealtimes, and whether help is properly attuned to the resident’s preferences. Every person has unique needs and preferences, and therefore the care provided should be in line with these needs to optimize eating performance [31, 40]. Moreover, unraveling the cause(s) is the starting point for further management of problems in clinical practice [41, 42]. The focus on unraveling causes can be explained by the fact that the majority of our participants were healthcare professionals. Healthcare professionals are trained to search for underlying causes and to find effective interventions to minimize consequences of problems they encounter in clinical practice [6]. The clusters that describe expressions of mealtime behavioral problems and consequences are in line with existing literature [3, 43].

### Strengths and limitations

A strength of this study is the diversity in backgrounds of the participating healthcare professionals in all phases of data collection. Also, the participants had experience with mealtime behavioral problems in both younger and older persons with dementia. The final concept map is based on diverse perspectives and therefore reflects a comprehensive picture of the problems as seen in clinical practice. Further, the overall response rates were sufficient and the stress value indicated a high goodness of fit with a value of 0.26 (average range 0.21-0.37) [23, 26, 27].

Although we included less than the 100 participants we aimed for, the information they provided was rich and therefore the contributions to the final concept map cover a broad range of content. This added to the reliability and validity of this study [27].

This study has some limitations. First, because the majority of participants were healthcare professionals, the perspectives from relatives and researchers may have been underrepresented, despite proactive recruitment of participants. The results should therefore be interpreted in that context, because the adequacy of the content is bounded by the source of input [26].

Second, interpretation of the results may be influenced by variations in cognitive style of the participants. However, this is a common flaw of the method, and integration in the conceptualization process is needed in further development of methods of group concept mapping [28, 44]. In group concept mapping, it is unknown to the researchers what the thoughts and interpretations of the participants were during data collection, especially during the sorting activity when they have to conceptualize ideas. This may have resulted in the risk that the research team not accurately reflected the content as seen through the eyes of the participants. On the other hand, Stoyanov et al. [44] suggest that a concept map can be considered as a group’s common cognitive contract that can consolidate individual differences and may serve as a tool for managing diversity in a group of participants.Compared with purely qualitative methods, the strength of group concept mapping lies in the combination of a qualitative and quantitative approach, using multivariate statistical analyses to conceptualize generated ideas from relevant stakeholders [28].

In conclusion, this group concept mapping study added to unraveling the complex nature of mealtime behavioral problems, as perceived by healthcare professionals, relatives, and researchers. We conclude that the overall concept cannot be defined easily and the different clusters overlap and interact with each other. The findings from this study indicate that ethical factors should be considered during identifying and management of mealtime behavioral problems. These ethical factors were present across all clusters, and refer to autonomy of the resident but also considerations about when to stop offering food and fluids. These factors are often overlooked, but this study emphasizes the importance. Therefore, it is recommended to comprehensively analyze all components in the management of mealtime behavioral problems, in particular being aware of ethical factors and align the provided care accordingly to residents with dementia. Multidisciplinary teams are advised to explicitly address ethical issues in team meetings when discussing treatment decisions. Based on the findings of this study, we recommend to view a situation from a broad perspective, and to not only focus on the behavior and related solutions, but also reflect on ethical considerations in general and themes such as respect for the autonomy of the resident versus sufficient intake of food and fluids. Further, it is recommended to actively involve family caregivers in the interpretation of behavioral symptoms, and subsequent care goals. With increased awareness of ethical factors, healthcare professionals may be better able to assess situations and to consider a moral deliberation in complicated cases or possible disagreements about the treatment decisions. We recommend further study on ethical factors and how they relate to mealtime behavioral problems. The search for balance between on the one hand autonomy of the resident and quality of life, and on the other hand sufficient nutrition is a continuous search and challenge. We recommend further research that explores the way professionals deal with ethical issues, such as the urge for weight maintenance while not forcing to eat, preserving the quality of life as much as possible while respecting the presumed wishes of the resident. As this study mainly describes the perspectives from healthcare professionals, it is recommended to further investigate the viewpoints of relatives and researchers in future research to further explore whether these views differ.

## Data Availability

The datasets used and/or analyzed during the current study are available from the corresponding author on reasonable request.
